# Cluster analysis for localisation-based data sets: dos and don’ts when quantifying protein aggregates

**DOI:** 10.3389/fbinf.2023.1237551

**Published:** 2023-11-24

**Authors:** Luca Panconi, Dylan M. Owen, Juliette Griffié

**Affiliations:** ^1^ School of Mathematics, Centre of Membrane Proteins and Receptors (COMPARE), Institute of Immunology and Immunotherapy, University of Birmingham, Birmingham, United Kingdom; ^2^ Department of Biochemistry and Biophysics, Stockholm University, Stockholm, Sweden

**Keywords:** cluster analysis, single molecule localisation microscopy (SMLM), protein aggregates, image quantification, bioinformactics, spatial point pattern (SPP)

## Abstract

Many proteins display a non-random distribution on the cell surface. From dimers to nanoscale clusters to large, micron-scale aggregations, these distributions regulate protein-protein interactions and signalling. Although these distributions show organisation on length-scales below the resolution limit of conventional optical microscopy, single molecule localisation microscopy (SMLM) can map molecule locations with nanometre precision. The data from SMLM is not a conventional pixelated image and instead takes the form of a point-pattern—a list of the x, y coordinates of the localised molecules. To extract the biological insights that researchers require cluster analysis is often performed on these data sets, quantifying such parameters as the size of clusters, the percentage of monomers and so on. Here, we provide some guidance on how SMLM clustering should best be performed.

## Introduction

Cellular processes heavily rely on the ability of key proteins to form aggregates, also called clusters. Immune cells for instance are regulated through subtle variations in signalling protein clustering characteristics. These clusters have now been shown to involve only a small number of proteins and range from 10 nm to 50 nm in size (Griffié et al., 2015). Until the development of super resolution microscopy (SRM), light microscopy, bound by the diffraction limit (>200 nm), was unable to resolve cells’ nanoscale architecture, including clusters. SRM in comparison encompasses imaging techniques with a spatial resolution below 200 nm. SMLM in particular achieves a resolution close to molecular scale (typically 10 nm) in cells (Lelek et al., 2021). It enabled, for the first time, the visualisation and quantification of protein nanoscale organisation including clusters, pores and filaments.

SMLM relies on the separation in time of fluorophores’ emission (i.e., blinks), which are collected over thousands of frames. Ideally, on each frame only a very small subset of well spatially separated fluorophores are emitting, allowing to extract from their diffraction limited point spread function their precise localisation. Therefore, the output of an SMLM acquisition does not consist of a conventional pixelated image, but rather of a spatial point pattern (SPP, i.e., scatter plot of collected localisations (x, y) in 2D (x, y, z) in 3D). Ultimately, it is the estimated uncertainty associated to every localisation that is often used as a proxy for spatial resolution. SPPs require totally different statistical tools for their analysis compared to pixelated images. Although relatively uncommon in microscopy, this type of dataset has been analysed for decades in fields such as ecology and epidemiology. This has provided a baseline for the development of a vast range of cluster analysis tools dedicated to SMLM (Khater et al., 2020). Here, we will discuss the dos and don’ts when analysing SMLM SPP for cluster quantification.

## Dos

### Pre-processing matters!

**FIGURE 1:**
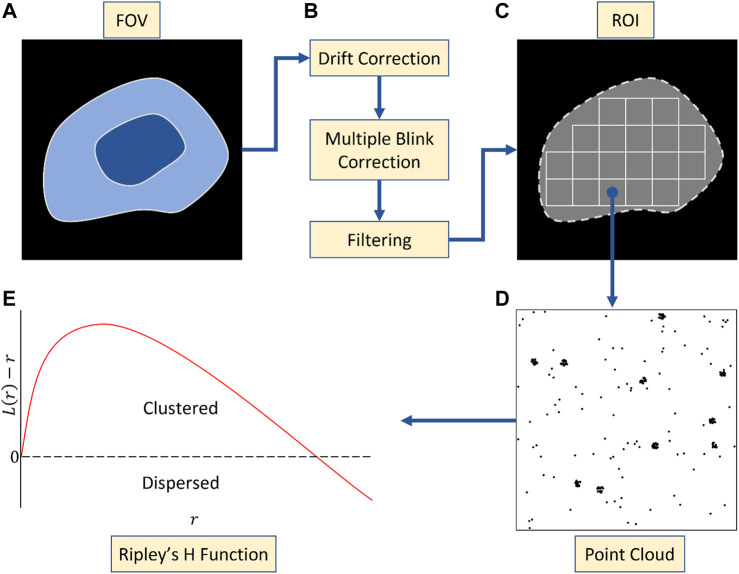
Pre-processing workflow. **(A)** Whole cell acquisition across a field of view (FOV). **(B)** Artefact correction following acquisition including drift correction, multiple blinking correction and filtering. **(C)** Segmentation of FOV into ROIs of fixed size. **(D)** Point clouds arising from one such ROI. **(E)** Use of the Ripley’s H function to determine the degree of clustering across the ROI. Non-negativity across radial values suggests clustering at that scale.

SMLM data sets require comprehensive curation before analysis (Figure 1). This includes drift correction and filtering badly localised emitters. Multiple blinking, which translates into localising the same fluorophore multiple times over the acquisition, also has to be addressed as it leads to self and artificial clustering (Annibale et al., 2011; Baumgart et al., 2016). In the case of PALM, there are 2 powerful existing strategies to account for multiple blinking. The first relies on the relative short timescale over which the reblinks tend to happen in order to merge them (Annibale et al., 2011). The second more recent solution is model-based and has been shown to provide artifact free point distribution for cluster analysis with unprecedented reliability (Jensen et al., 2022). In both cases, multiple blinking correction should be included in the analysis pipeline upstream of the cluster analysis. When it comes to STORM however, the issue has not been fully addressed and remains an open avenue of research (Bohrer et al., 2021). A good practise however remains to merge blinks that extend over multiple frames.

Multiple blinking is not the only pre-processing requirement for most cluster analysis tools. A field of view is typically made of millions of localisations, with strong variations in overall point density (e.g., in and outside the cell) and clustering (e.g., sizes), for which most methods fail to provide accurate clustering descriptors. This results from widespread cluster analysis methods [e.g. DBSCAN (Ester et al., 1996), localised Ripley’s K (Owen et al., 2010)] reliance on fixed user parameters which cannot fit all noise and clustering variations in the same ROI as well as across ROIs. The computational cost of handling millions of points is also a key limitation, apart from graph-based methods specifically repurposed for large data sets (e.g., whole cell, field of view) (Levet et al., 2015). Both factors indicate the overall need to define regions of interest (ROI) in which the number of points does not limit the computation and the clusters within the picked ROI display similar characteristics. In cases where broad cluster density/size range (within the same ROI or across the data sets) as well as uneven background cannot be addressed with ROI picking, a Bayesian-based cluster analysis tool will typically provide more reliable results as it picks the best parameters pair, in regard to a realistic model on protein aggregation in cells, independently for each ROI (Rubin-Delanchy et al., 2015; Griffié et al., 2016). In all cases, ROI should also be associated to edge correction strategies. Most cluster analysis tools now include edge correction directly in the analysis pipeline (e.g., symmetry, framing) but the user must be aware of which strategy is implemented to provide an ROI of bigger size for instance if required.

### Is cluster analysis needed?

Randomness in biology is hardly ever uniform, but rather consist of heterogenous distribution in which each point has an equal probability to be at any location in the ROI, also called complete spatial randomness (CSR). CSR can manifest in SPPs in which clustering like structures can be seemingly present. When looking for clusters, it is therefore crucial to avoid fitting your data and analysis to your prior and hypothesis. An efficient way to differentiate a CSR from a clustered distribution is to use Ripley’s K curve analysis (Kiskowski et al., 2009; Owen et al., 2010). These curves are calculated by averaging over the point population and ROI, the local density for various scales and are today implemented as pre-made functions in most coding interfaces. It provides a very robust statistical tool to differentiate with high confidence if the protein distribution studied is CSR or clustered and hence suited to further cluster analysis (assuming multiple blinking has been accurately corrected for). Ripley’s K curves however do not provide cluster identification and visualisation or detailed information on clusters’ composition and sizes. In cases where multiple blinking cannot be easily addressed (e.g., STORM), the clustering landscape may still be accessible through experimental SMLM approaches as suggested by Arnold et al. (2020).

Finally, most available cluster analysis tools focus on circular shapes and are overall unsuitable for multiscale or shape independent segmentation. As a result, virtually all available cluster analysis tools are unsuitable to quantify filamentous structures, and only some can accommodate for elongated aggregates or rings (Ester et al., 1996; Pike et al., 2020). Whilst novel dedicated approaches have started to emerge for filamentous mesh quantification in SMLM data sets (Peters et al., 2018), it overall remains an active topic of research.

### Optimise and report user defined parameters

There are a wide variety of cluster analysis algorithms available, even when only considering those that have been tested and validated for use on SMLM data (Khater et al., 2020). While they all have advantages and disadvantages compared to each other, one property that almost all share is the use of user-defined analysis settings. These are numbers that the user must enter into the algorithm to dictate what kind of structural features should be highlighted in the data. A common necessity is for two parameters—one somehow related to the spatial scale of objects of interest and the other related to the density of points within the clusters, i.e., is the user looking for big or small clusters and are they looking for sparse or dense clusters? Naturally, the choice of these parameters can strongly influence the output of the analysis. In some sense, there is no right or wrong answer to the choice because which clusters in the data are most relevant depends on the biological questions being asked. A minimum requirement is therefore to simply report the choice of parameters when describing the method so the results can be reproduced. However, if something about the data is known *a priori*, it is possible to optimise the choice of analysis parameters. For example, using a success or performance metric, analysis can be performed while scanning the values of analysis parameters and the best performing parameters chosen for continued use. This can be done especially if one can simulate data that closely recapitulates the experimental case (Nieves et al., 2023) or if prior knowledge can be summarised about the expected clustering properties (Rubin-Delanchy et al., 2015). Furthermore, Nieves et al. (2023) provides a detailed performance assessment on the vast majority of the cluster analysis tools described in this minireview in order to help user identifying which algorithm may be best suited to their data sets.

## Don’ts

### Analyse blindly

Most researchers will typically seek a completely automated analysis pipeline. This not only saves time but there is also a perception that it reduces user bias if a human makes no decisions within any particular analysis. While these are worthy goals, SMLM clusters analysis algorithms are not yet capable enough to warrant this level of confidence. A frequent occurrence in SMLM are unexpected features in the data sets. These might be real but rare biological structures, misplaced artefacts of the sample such as fiducial markers or other contaminants or unexpected artefacts of the imaging and analysis such as cell edges, uncorrected drift or sparse data. Inputting such data sets into the algorithms will produce meaningless results and bias biological conclusions. All images undergoing analysis should therefore be inspected visually to ensure the data structure is compatible with the proposed analysis. In addition, several algorithms exist which can help the user locate and assess potential data artefacts and allow them to therefore perform analysis with confidence. These include HAWKMAN (Marsh et al., 2021) and SQUIRREL (Culley et al., 2018).

### Treat results as absolute quantification

SMLM data sets are artifact prone. This results both from the sample preparation stage and the processing stage. For sample preparation, most SMLM acquisitions rely on immunolabelled sample or transfection. Both strategies come with sampling issues. At the processing stage, STORM does not have reliable strategies to account for multiple blinking to date and, for both PALM or STORM, a subset of the fluorophore population will not be detected at all. For all these reasons any quantification extracted from cluster analysis tools should be treated as relative rather than absolute. Typically, this translates into using the term “localisation” when talking about the clusters’ composition, rather than protein. Cluster analysis tools are thus suited for relative comparison in between conditions rather than the description of the exact protein composition of identified aggregates. If absolute quantification is required for the biological issue at hand, there are today emerging experimental and statistical means to by-pass this issue with PAINT (Simoncelli et al., 2020) but they remain very low throughput.

## Discussion

Overall, SMLM is a powerful tool for obtaining the precise locations of membrane proteins on the cell surface. However, to derive biologically meaningful conclusions such as describing the nanoscale clustering of those proteins, that data must be processed and analysed. We propose that users adopt a standardised analysis pipeline for their analysis which is broken down into a number of stages. First, data curation in which imaging artefacts such as drift and multiple-blinking can be corrected and data formatted into standardised ROIs. Second, data validity. A visual inspection of the ROIs to identify artefacts and the use of image quality algorithms will ensure data passed down the pipeline is valid. In particular Ripley’s K-function should be used to confirm the presence of clustering. Third: Cluster analysis. Using prior or preliminary data, data analysis parameters should be optimised and reported and the results subjected to a secondary visual inspection. Finally, interpretation. Keeping in mind that none of the above steps can be completed perfectly. Each will add some uncertainty and bias to the final output and will sit on top of the artefacts arising during sample preparation, imaging and localisation. As a general rule therefore, users should be wary of treating outputs as absolute and SMLM cluster analysis is most powerfully utilised to compare between experimental conditions.
